# 176. Antibiotic Resistance Patterns, Seasonality, and Correlation with the Influenza Season in the United States: A Multicenter Evaluation Reveals Surprising Association Between Influenza Season and Gram Negative Pathogens

**DOI:** 10.1093/ofid/ofab466.176

**Published:** 2021-12-04

**Authors:** Amine Amiche, Heidi Kabler, Janet Weeks, Kalvin Yu, Vikas Gupta

**Affiliations:** 1 Sanofi Pasteur, Dubai, Dubai, United Arab Emirates; 2 Becton, Dickinson and Company, Franklin Lakes, New Jersey

## Abstract

**Background:**

Influenza infection may affect bacterial transmission dynamics and seasonality of antimicrobial resistance (AMR). There is a paucity of data on the association of influenza season and AMR rates. We aimed to describe trends of AMR and their correlation with the influenza season in ambulatory and inpatient settings in the United States (US).

**Methods:**

We used the *BD Insights Research Database* (Franklin Lakes, NJ USA) to identify 30 day non-duplicate isolates collected from patients >17 years old with susceptibility profile of Gram-negative (GN) (Enterobacterales (ENT), *P. aeruginosa* (PSA), *A. baumannii spp.* (ACB), and *S. maltophilia* (Sm)) and Gram-positive (GP) pathogens (*S. aureus* (SA), and *S. pneumoniae* (Sp)) in up to 257 US healthcare institutions from 2011-19. We defined the outcomes as rates per 100 admissions and % of non-susceptibility (NS), stratified by community and inpatient settings, resistance type (resistance to carbapenem (Carb-NS), quinolone (FQ-NS), macrolide (Macr NS), penicillin (PCN NS), and extended spectrum cephalosporin (ESC NS)) and isolate origin (respiratory and non-respiratory). Influenza data were presented as the % of positive laboratory tests. We used descriptive statistics and generalized estimating equations models to evaluate the monthly trends of AMR outcomes and correlation with the influenza season.

**Results:**

We identified 16 576 274 confirmed non-duplicate pathogens, of which 154 841 were GN Carb-NS, 1 502 796 GN FQ-NS, 498 012 methicillin resistant SA (MRSA), and 44 131 Macr-NS, PCN-NS, and ESC-NS Sp. Among the Carb-NS pathogens, Influenza rate was correlated with % ACB-NS [β= 0.205, p< .001]. In the FQ-NS group, influenza was associated with overall % ENT-NS [β= 0.041 p< .001] and % PSA-NS [β= 0.039, p = .015]. For the GP pathogens, all Sp. rates were correlated with increased influenza positivity % (See Table). Only MRSA rates of respiratory source were associated with influenza [β= .066, p=.028].

Summary of Multivariate regressions of AMR and % Flu by Source and Setting (controlling for hospital level factors): 2011-2019

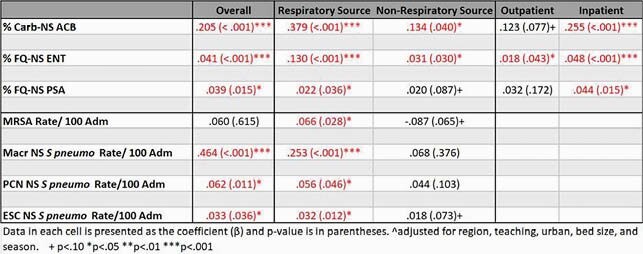

Data in each cell is presented as the coefficient and p-value is in parentheses. ^adjusted for region, teaching, urban, bed size, and season. + p<.10 *p <.05 **p <.01 ***p <.001

**Conclusion:**

Our study revealed surprising association between influenza epidemics and GN resistance and corroborated the evidence of correlation between respiratory GP and influenza infections. These insights may help inform targeted antimicrobial stewardship initiatives during influenza season.

**Disclosures:**

**Amine Amiche, PhD**, **Sanofi** (Employee, Shareholder) **Heidi Kabler, MD**, **Sanofi Pasteur** (Employee) **Janet Weeks, PhD**, **Becton, Dickinson and Company** (Employee) **Kalvin Yu, MD**, **BD** (Employee) **Vikas Gupta, PharmD, BCPS**, **Becton, Dickinson and Company** (Employee, Shareholder)

